# Interactions of the Greater Ontong Java mantle plume component with the Osbourn Trough

**DOI:** 10.1038/srep37561

**Published:** 2016-11-21

**Authors:** Guo-Liang Zhang, Chao Li

**Affiliations:** 1 Key Laboratory of Marine Geology and Environment, Institute of Oceanology, Chinese Academy of Sciences, Qingdao 266071, China; 2 Laboratory for Marine Geology, Qingdao National Laboratory for Marine Science and Technology, Qingdao, 266061, China; 3 Institute of Geology Chinese Academy of Geological Sciences, Beijing 100037, China

## Abstract

The Ontong Java-Manihiki-Hikurangi plateau (OJMHP) is considered to have originated from a starting mantle plume, and have been rifted apart by two spreading ridges. However, the ages of these spreading ridges and their possible interactions with the presumed mantle plume are unclear. The Manihiki-Hikurangi plateau has been rifted apart by the Osbourn Trough which formed the southwestern Pacific crust to the east of the Tonga-Kermadec trench. Here we report Pb-Hf-Os isotopes of the basaltic crust (Site U1365 of IODP Expedition 329) formed by the Osbourn Trough. Linear regression of Re-Os isotopes results in an age of 103.7 ± 2.3 Ma for Site U1365 basalts, indicating that the Manihiki-Hikurangi plateau was rifted apart by the Osbourn Trough with a spreading rate of ~190 mm/yr. The superfast spreading rate supports the Osbourn as an abandoned segment of the early Pacific spreading ridge, which initially overlapped with the giant starting plume. Moreover, the Pb-Hf isotopes of some of Site U1365 basalts show distinct differences from those of the Pacific mid-ocean ridge basalts, while they are similar to the basalts of the Ontong Java and Manihiki plateaus. We suggest that the OJMHP mantle plume components has been involved by the Osbourn spreading center.

The Ontong Java, Manihiki and Hikurangi plateaus have been shown to have been a joint plateau (the Greater Ontong Java, or OJMHP), which represents the biggest large igneous province (LIP) on the Earth^1^,^2^,^3^. This joint plateau (OJMHP) is broadly considered as the result of a starting mantle plume (plume head) originating from the core/mantle boundary during a short volcanic episode (125–117 Ma)^4^,^5^,^6^,^7^. However, the lack of a subsequent hotspot track (representative of plume tail) challenges its origin as a starting mantle plume^8^,^9^.

The OJMHP was rifted apart by two spreading centers, namely the Ellice basin spreading center separating the Ontong Java and the Manihiki, and the Osbourn Trough (Fig. 1) separating the Manihiki and the Hikurangi^1^,^10^. The two spreading centers were active during the Cretaceous normal superchron (125–84 Ma), hence, their spreading history and possible link to the OJMHP remain unclear^11^,^12^,^13^. The Osbourn Trough (Fig. 1) is considered to have rifted apart the Manihiki-Hikurangi plateau and formed the southwestern Pacific basin (Fig. 1) between the two plateaus^1^,^11^,^14^. If there were continued mantle plume activities after the OJMHP, it would have affected the mantle source of the Osbourn Trough via plume/ridge interactions, e.g., interactions of the Iceland hot spot with the mid-Atlantic ridge^15^ and the Easter hotspot with the East Pacific Rise^16^. Additionally, the lithosphere of the OJMHP would also have been detached and contaminated the asthenospheric mantle during the rifting processes, which could have been sampled by the Osbourn Trough magmatism. Hence, the Osbourn spreading history and geochemistry of basalts formed by the Osbourn Trough are crucial to test if the mantle plume components of the OJMHP have interacted with the Cretaceous Osbourn Trough.

## Samples and Results

**Figure 1:**
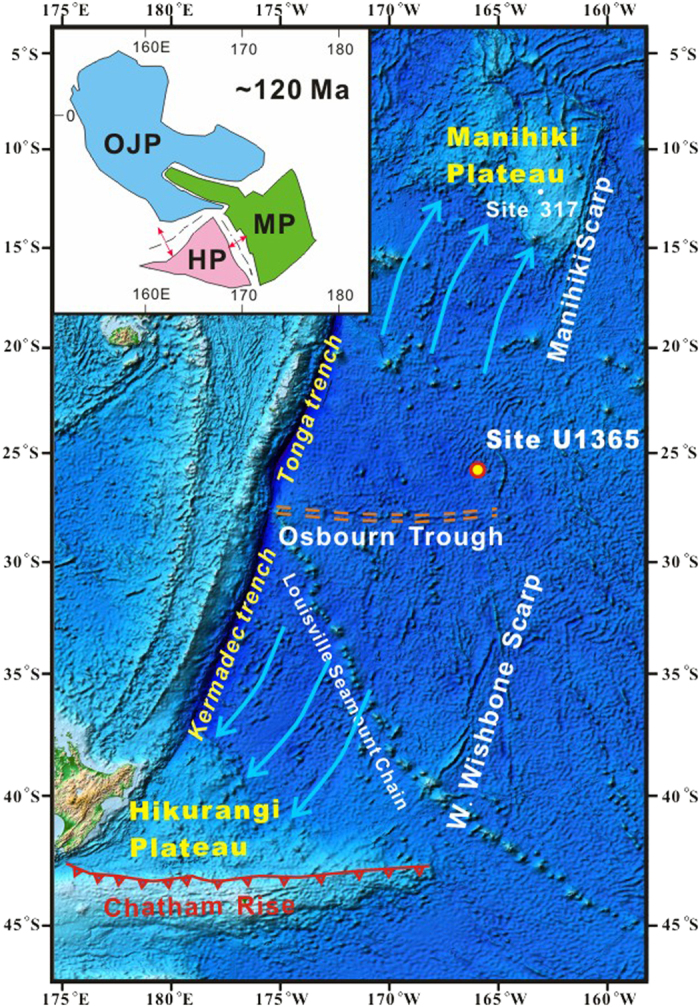
Map of southwestern Pacific basin and location of IODP Expedition 329 Site U1365. Bathmetric data are from http://www.geomapapp.org/ and map was produced using the GeoMapApp software (Version 3.6.0) (http://www.geomapapp.org/ArchiveDownloads.html). Inset shows the Greater Ontong Java (or OJMHP) at ~120 Ma which is modified according to Fig. 3 of Taylor^1^, dashed lines, rifting spreading centers, red arrows, spreading direction.

**Figure 2:**
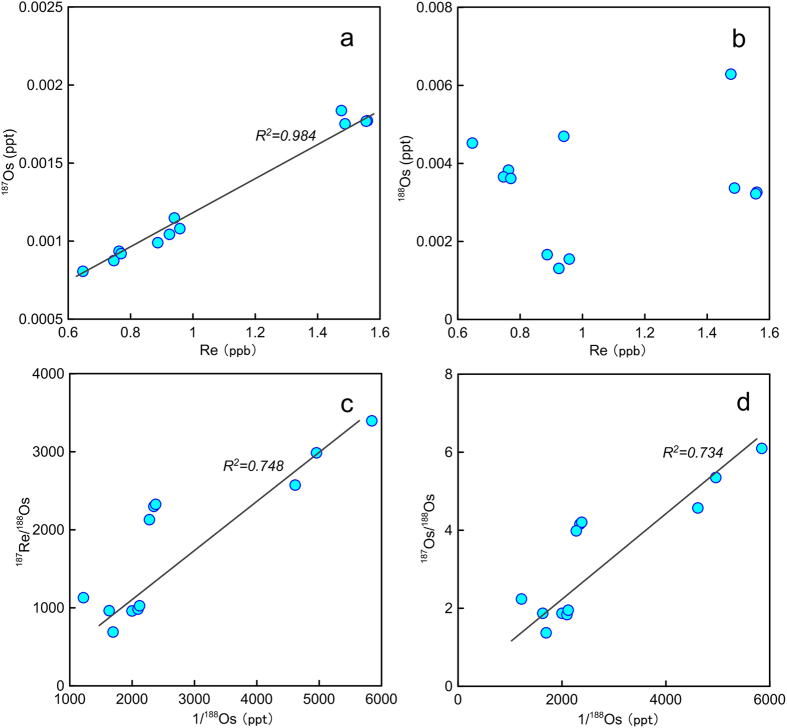
Plots showing relationships of Re vs. (**a**) ^187^Os and (**b**) ^188^Os, and 1/^188^Os vs. (**c**) ^187^Re/^188^Os and (**d**) ^187^Os/^188^Os.

**Figure 3:**
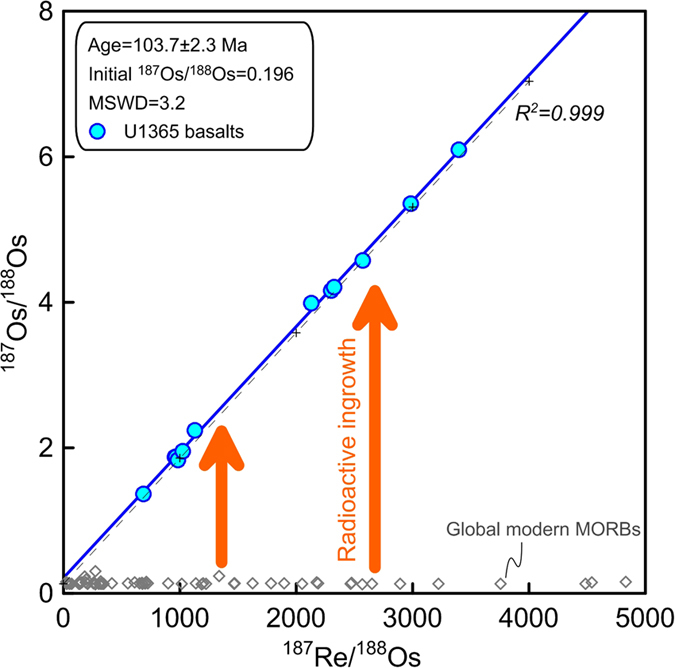
Plot of ^187^Re/^188^Os vs. ^187^Os/^188^Os showing apparent isochron age of Site U1365 basalts. The regression age is calculated using the ISOPLOT program. The analytical errors are smaller than the symbol. The dashed line with crossed symbols is the calculated isochron of 103.7 with an initial ^187^Os/^188^Os of 0.133. Global modern MORBs (EPR, mid-Atlantic ridge and Indian ridge) data are from Gannoun *et al.*^21^, Escrig *et al.*^45^ and Yang *et al.*^46^.

**Figure 4:**
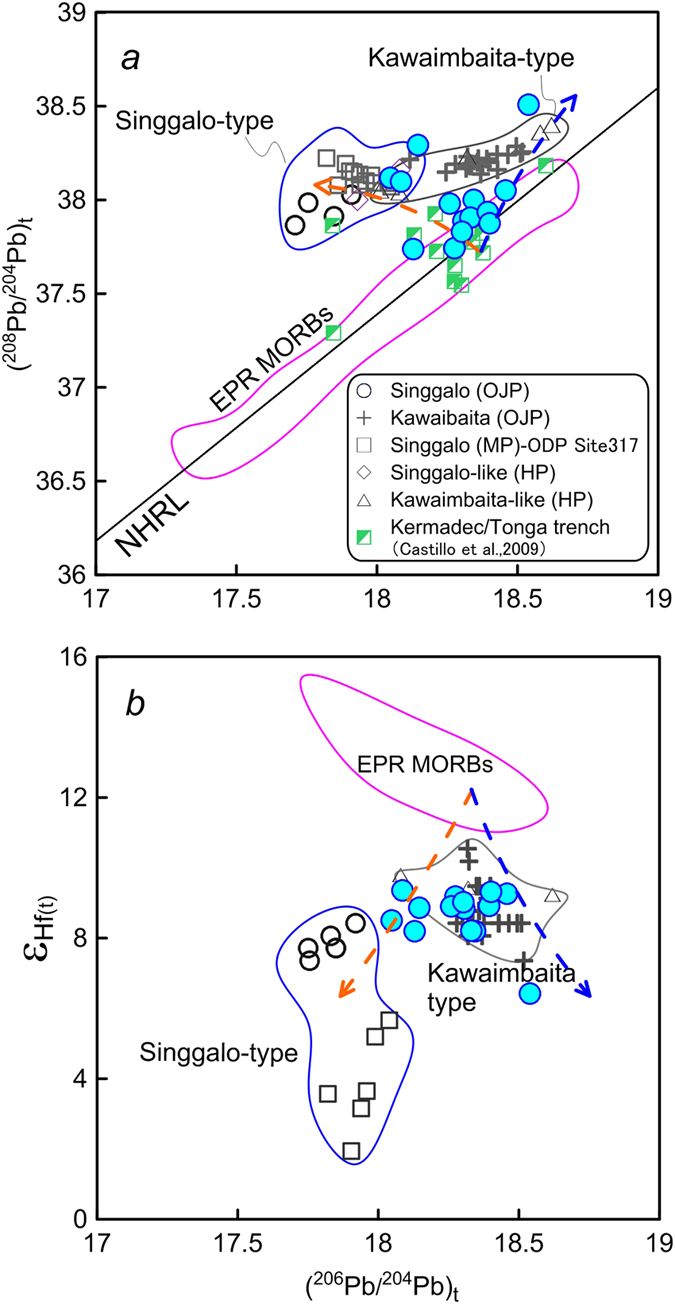
Plots of (^206^Pb/^204^Pb)_t_ vs. (**a**) (^208^Pb/^204^Pb)_t_ and (**b**) εHf_t_ for Site U1365 basalts. Legend of Site U1365 basalts is as in Fig. 2. The orange and blue dashed lines indicate estimated mixing trend with a mantle source of Singgalo and Kawaimbaita, respectively. Data source for comparison: East Pacific Rise (EPR) MORBs, http://www.earthchem.org/petdb; Data for the Singgalo type basalts (Ontong Java and Manihiki) are from Tajada *et al.*^7^,^33^, Hoernle *et al.*^2^ and Timm *et al.*^3^; Data for the Kawaimbaita type basalts of Ontong Java are from Tajada *et al.*^7^,^33^. Data for Hikurangi plateau (Kawaimbaita-like and Singgalo-like) are from Hoernle *et al.*^2^. The North Hemisphere Reference Line (NHRL) is according to Hart^47^.

Basalt samples from Site U1365 of IODP Expedition 329, which is located ~250 km to the north of the Osbourn Trough (Fig. 1), were analyzed for Hf-Pb-Os isotopes in this study. Only the fresh samples based on thin section observations are selected for analyses. Results of Re-Os isotopes, Hf and Pb isotopes in this study are shown in Supplementary Table. These basalt samples have very low but variable contents of Os (0.81–6.3 ppt), while contents of Re are less variable (0.65–1.56 ppb), indicating strong Re/Os fractionation during magmatic processes (Fig. 2). These basalt samples have wide ranges of ^187^Re/^188^Os (689–3395) and ^187^Os/^188^Os (1.368–6.098) (Supplementary Table). Although these samples have (^206^Pb/^204^Pb)_t_ (18.05–18.54) within the range of the Pacific normal mid-ocean ridge basalts (N-MORBs) (Fig. 3), several samples are decoupled from the East Pacific Rise (EPR) N-MORBs on the plot of (^208^Pb/^204^Pb)_t_ vs. (^206^Pb/^204^Pb)_t_ (Fig. 4a). Their εHf_t_ are also distinctly lower than those of EPR N-MORBs (Fig. 4b).

## Re-Os Systematics and Age

The concentration of Re tends to increase with decreasing MgO and Al_2_O_3_ and increasing SiO_2_, TiO_2_, Nb and La (Fig. S1), which are consistent with the moderate incompatibility of Re during magma fractionation as suggested by previous studies^17^,^18^. These correlations corroborate that these samples are fresh and exclude the ‘nugget’ effect^19^, which is further excluded by replicated analyses of Re (see Supplementary Table). Unlike Re, Os is strongly compatible during mantle melting and magma fractionation^20^, which can result in an elevated ratio of ^187^Re/^188^Os in this study. The low concentrations of Os in this study and their lack of correlation with concentrations of Re (Fig. 2b), MgO and Ni (Fig. S2) indicate a role of either mantle source heterogeneity or ‘nugget’ effects, however, replicated analyses of Os also indicate a negligible ‘nugget’ effect (Supplementary Table). Mixing of intraplate magma with ancient oceanic crust with a high ^187^Os/^188^Os ratio might increase the ratio of ^187^Os/^188^Os. However, the depleted trace element patterns of these samples and geologic setting of Site U1365 point to MORBs and exclude them as ocean island basalts^13^, thus, contamination of ancient crust during magma processes is unlikely. Because Re and Os behave differently during magma processes, the highly variable concentrations of ^187^Os and their positive correlation with Re (Fig. 2) are related to post eruption radiogenic ingrowth. The extremely low abundance of ^188^Os indicates low initial ^187^Os, thus, the post-eruption radiogenic ^187^Os resulted from the high ^187^Re/^188^Os ratios accounts for a majority of the total ^187^Os abundance.

These samples show excellent correlation and linearity (R^2^ of 0.999) between ^187^Re/^188^Os and ^187^Os/^188^Os (Fig. 3). Samples in this study have much higher ratios of ^187^Os/^188^Os than those of global modern MORBs, which cannot be related to mantle source heterogeneity. The positive correlation of ^187^Os/^188^Os vs. 1/^188^Os was resulted from radiogenic ingrowth in these samples with variable initial ^187^Re/^188^Os ratios. The well-correlated ratios of ^187^Os/^188^Os vs. ^187^Re/^188^Os indicate radiogenic ingrowth of ^187^Re in a closed system. The linearity (R^2^ of 0.999) also indicates negligible influences of variation in initial ^187^Os/^188^Os ratios. Linear regression of ^187^Re/^188^Os vs. ^187^Os/^188^Os results in an apparent age of 103.7 ± 2.3 Ma for Site U1365 basalts (Fig. 3). An initial ^187^Re/^188^Os ratio of 0.196 is obtained for this suite of samples based on the linear regression, which is much higher than those of EPR N-MORBs (Fig. 3), e.g., ~0.133 ± 0.009 in average based on Gannoun *et al.*^21^ (this average value would be 0.1327 after correction to 103.7 Ma according to the average Re/Os ratio of peridotite in Liu *et al.*^22^). The calculated initial ^187^Os/^188^Os ratio of 0.196 is much higher than the global modern MORBs, implying a mantle source with long-term enrichment of Re relative to Os. The regression age from Re-Os isotopes is interpreted to reflect the formation age of Site U1365 basalts.

## Nature and Spreading History of the Osbourn Trough

The cessation age and spreading rate of the Osbourn Trough have long been debated because the Osbourn Trough was active during the Cretaceous normal superchron (125–84 Ma)^11^,^12^,^13^. Estimated cessation age of the Osbourn Trough varies between 105 Ma–71 Ma^11^,^12^,^14^,^23^,^24^. The Osbourn Trough has been shown to have a medium to slow spreading rate (60–80 mm/yr) according to the morphology of the fossil ridge^11^, which, however, could also have been a fast ridge with decreasing rate before cessation. It was also considered as a fast ridge which belongs to a segment of the early Pacific spreading center^12^, which could have originally overlapped with the OJMHP^25^,^26^ and rifted apart the Manihiki-Hikurangi plateau immediately after its formation.

Based on the age of 103.7 Ma for the basalt of Site U1365, if the rifting of the joint Manihiki-Hikurangi plateau by the Osbourn spreading center occurred immediately after its formation (e.g., ~119 Ma)^1^, the Osbourn Trough would have a minimum full spreading rate of 190 mm/yr according to the distance of ~1500 km between Site U1365 and the Manihiki plateau. This spreading rate exceeds that of the fastest southern East Pacific Rise (160 mm/yr)^27^. This also indicates that the medium to low spreading rate based on the fossil ridge morphology^11^ could have resulted from the decreases in spreading rate before cessation.

This superfast spreading rate supports the prediction that the Osbourn Trough belongs to an extinct section of the Cretaceous Pacific-Phoenix ridge^12^,^23^. Thus, the joint Manihiki-Hikurangi plateau would have overlapped with the Cretaceous Pacific-Phoenix ridge during its initial formation. Moreover, if a constant spreading rate is assumed for the Osbourn Trough before cessation, it would have ceased at ~101 Ma according to its distance from Site U1365. This age is in concert with the collision time of the Hikurangi plateau with the Chatham rise (Fig. 1) at ~100 Ma^14^, which is considered as the cause of cessation of the Osbourn spreading^12^,^14^.

## Interactions with the OJMHP Mantle Plume Component

The initial ^187^Os/^188^Os ratio of 0.196 calculated for this suite of samples is much higher than those of the Pacific N-MORBs (Fig. 3) (0.126 to 0.148, based on Gannoun *et al.*^21^). The ^187^Os/^188^Os ratios in this study are systematically higher than the isochron line of 103.7 Ma with an initial ^187^Os/^188^Os ratio of 0.133 (Fig. 3), which corroborates that the samples in this study have systematically higher (^187^Os/^188^Os)_t_ than the Pacific N-MORBs. Such high initial ^187^Os/^188^Os ratios (0.196) have never been observed in the EPR MORBs, indicating secular enrichments of Re relative to Os in the mantle source or contamination from a mantle source having long-term Re-enrichment relative to Os. Because Re and Os are not sensitive to metasomatism caused by melts or fluids^25^, potential mantle sources enriched in Os isotope (with high ^187^Os/^188^Os) would not have been derived from metasomatized oceanic/continental lithospheric mantle. Magmatic processes can cause strong enrichment of Re relative to Os^28^,^29^, thus, a mantle source with anomalous high (^187^Os/^188^Os)_t_ could have been related to contamination of crustal materials, e.g., recycled continental/oceanic crust and terrigenous sediments. Origin of such high initial ^187^Os/^188^Os ratio could be either recycled components imbedded in depleted asthenosphere, or contamination from an external component during mantle melting/evolution. However, the unusually high initial ^187^Os/^188^Os ratio of Site U1365 basalts relative to the EPR MORBs (and also global MORBs) imply that it is unlikely resulted from melting of normal asthenosphere under the spreading ridge.

Based on the unique geologic setting of Site U1365, contamination of a mantle source with high (^187^Os/^188^Os)_t_, e.g., through interactions with nearby mantle plumes, should be evaluated. The Louisville seamount chain intersects the Osbourn Trough at its western end (Fig. 1). Previous studies showed that the Louisville seamount chain might have influenced the Osbourn Trough magmatism through plume/ridge interactions^30^. However, according to the cessation time at ~101 Ma for the Osbourn Trough the Louisville seamount chain would not have interacted with the Osbourn Trough magmatism, because the oldest seamount that intersects the Osbourn Trough is ~79 Ma^31^. A recent study showed homogeneous and normal compositions of the LSC basalts in Os isotope ((^187^Os/^188^Os)_t_ (0.1245–0.1314)^32^, which further rules out the possibility of contamination from the Louisville seamount chain.

The mantle plume components of OJMHP are potential sources that have contaminated the lavas formed by the Osbourn Trough^15^. Based on the exposed crust in Solomon islands, the Ontong Java plateau is composed of two isotopically distinct groups of volcanic rocks, the Kawaimbaita-Kroenke type and the Singgalo type^7^,^33^. An increasing number of studies show that these two groups are widely distributed in the Ontong Java, Manihiki and Hikurangi plateaus^2^,^3^. The Singgalo type basalts have lower ^206^Pb/^204^Pb and εHf (Enriched Mantle 1- or EM1-like component) than those of the Kawaimbaita-Kroenke type (Fig. 4)^7^,^33^,^34^. The basalts of both types from the Ontong Java plateau have exceptionally high (^187^Os/^188^Os)_t_ (e.g., up to 0.4 for Singgalo type and 0.26 for Kawaimbaita type), which are considered to have been derived from recycled continental crust^33^. Publication on Re-Os isotopes of the Manihiki and Hikurangi basalts has long been lacking. However, a recent study of Schaefer^35^ reported high ratios of (^187^Os/^188^Os)_t_ (up to >0.17) for the Manihiki low-Ti basalts (ranging isotopically from the Kawaimbaita-Kroenke type on the OJP to a HIMU component).

The OJMHP Singgalo/Kawaimbaita type components with high (^187^Os/^188^Os)_t_ ratios are likely the potential contaminant in the source of Site U1365 basalts. Additionally, the low-Ti Kawaimbaita basalts from Manihiki were also shown to have high (^187^Os/^188^Os)_t_ ratios^35^. Site U1365 basalts have different (^206^Pb/^204^Pb)_t_ and distinct εHf_t_ from EPR N-MORBs and their initial ^187^Os/^188^Os ratio is between the OJMHP samples and EPR N-MORBs, indicating that the OJMHP mantle source might have contributed to melts of the Osbourn spreading center. Contamination of a N-MORB type mantle source by the OJMHP mantle source is sensitive in (^187^Os/^188^Os)_t_ ratios because of the higher Os contents in plateau samples (>20 ppt in average) than Site U1365 samples.

Site U1365 basalts have variable (^208^Pb/^204^Pb)_t_ ratios for a given ratio of (^206^Pb/^204^Pb)_t_ from typical EPR MORBs to the domain of OJMHP basalts (Fig. 4a). These samples also deviate from the EPR MORBs and extend to the range of OJMHP basalts on the plot of (^206^Pb/^204^Pb)_t_ vs. εHf (Fig. 4b). These also imply contamination of the OJMHP mantle source on the Site U1365 basalts. One sample in this study has relatively high (^206^Pb/^204^Pb)_t_ and (^208^Pb/^204^Pb)_t_ and low εHf_t_, which is similar to the Kawaimbaita basalts of the Ontong Java plateau.

The oceanic crust formed by the Osbourn spreading center is currently subducting into the Kermadec-Tonga trench (Fig. 1). Castillo *et al.*^36^ reported Pb isotopes of basalt samples dredged from the incoming plate of Kermadec-Tonga trench. The data on the N-MORBs reported by Castillo *et al.*^34^ are plotted in Fig. 4 for comparison. Similar to Site U1365 basalts, several samples reported by Castillo *et al.*^36^ also deviate from the EPR MORBs and extend towards the OJMHP domain on plot of (^208^Pb/^204^Pb)_t_ vs. (^206^Pb/^204^Pb)_t_, one of which is solely in the domain of Singgalo-type basalts (Fig. 4). These data and the new data in this study jointly support the contamination of the mantle source of Osbourn spreading center by the OJMHP mantle plume component.

One likely explanation for the contamination of Site U1365 basalts by the OJMHP mantle plume component is plume/ridge interations^15^. The volcanism caused by this starting mantle plume is distributed far more than on these plateaus. The crust in the Nauru basin and the Pacific crust to the east of the Mariana trench also show similar formation age and geochemistry to the OJMHP basalts^37^. This is analogous to effects of the Iceland mantle plume on the mid-Atlantic ridge. The effects of the OJMHP mantle plume on the Pacific spreading centers are expected to be stronger than the Iceland mantle plume on the mid-Atlantic ridge, because the superfast spreading ridge would have caused more consumption of mantle plume material for a given period.

Detailed sampling and age-dating studies on the Ontong Java, Manihiki and Hikurangi plateaus showed that they were formed in at least two stages, 125–117 Ma and 96–88 Ma, respectively^2^,^34^. This is evidence for continued volcanic activities in the Manihiki and Hikurangi plateaus after the major volcanic stage (125–117 Ma) on the OJMHP. It is difficult to infer the location of the presumed plume center over the two volcanic stages, however, the geochemical similarities of basalts formed by the two stages indicate a common mantle source^7^,^34^. Thus, the volcanic activities on the OJMHP have continued to later than 100 Ma. However, it is not clear if these volcanic activities after the major stage of OJMHP formation are related to the presumed mantle plume tail after the starting mantle plume melting. Additionally, there is lack of clear seamount chains between the Osbourn Trough and the Manihiki and Hikurangi plateaus, which indicate flows of mantle plume components to the ridge.

A starting mantle plume has been supposed to spread horizontally to a diameter exceeding 2000 km in the shallow mantle^38^,^39^, which melts and produces a giant oceanic plateau in a short period (i.e., 5 Myr). Both the Kawaimbaita- and the Singgalo-type basalts are distributed on the Ontong Java, Manihiki and Hikurangi plateaus, corroborating the widespread distribution of plume component in the southwestern Pacific. The starting mantle plume materials might have stagnated in the asthenospheric mantle after formation of the overlying oceanic plateau. If the residual mantle plume material of the OJMHP has stagnated in the asthenosphere, it would have been entrained by the Osbourn spreading center and contributed to the MORB magmas of the Osbourn Trough.

## Conclusion

We report Pb-Hf-Os isotopes of Site U1365 basalts from IODP Expedition 329 to investigate the mantle source nature and spreading history of the Cretaceous Osbourn Trough. The basalt samples show much higher ^187^Os/^188^Os ratios than global modern MORBs that is obviously caused by post-eruption radiogenic ingrowth. The good linearity (*R*^*2*^ of 0.999) of ^187^Os/^188^Os vs. ^187^Re/^188^Os gives an apparent regression age of 103.7 ± 2.3 Ma for Site U1365 basalts. This age is consistent with the prediction that the Manihiki-Hikurangi plateau was rifted apart by the Osbourn spreading center with a superfast rate (~190 mm/yr). This also indicates a cessation time of ~101 Ma for the Osbourn trough if a constant spreading rate is assumed, which is in concert with the time of collision between the Hikurangi plateau and Chatham Rise. Moreover, Site U1365 basalts have an initial ^187^Os/^188^Os of 0.196 based on regression of ^187^Os/^188^Os vs. ^187^Re/^188^Os, which is much higher than those of EPR MORBs. The Pb-Hf isotope compositions of Site U1365 reflect contamination from mantle plume components from the OJMHP. We propose that the mantle source of the Cretaceous Osbourn spreading ridge could have been contaminated by the detached lithosphere of the OJMHP during its rifting.

## Methods

### Pb-Hf isotope analyses

Sample powders used for analyses of Pb-Hf isotopes in this study were leached in hot 6 N HCl for ~30 minutes to remove any potential seawater contamination, rinsed in water purified in a Milli-Q reverse osmosis system to remove any residual traces of acid and dried. For analyses of Pb isotopes, sample powders were dissolved with 1 mL HNO_3_ plus 4 mL HF. Then, the dissolution was dried and added with HNO_3_ for three times until there was no HF left. The dissolution was dried and dissolved by 1 mol/L HBr and transferred to the microtube for centrifugation. The AG1–8 anion resin was used for Pb separation using the standard procedure. Pb isotopes were analyzed using a Nu instrument MC-ICP-MS at Institute of Geology Chinese Academy of Geological Sciences (IGCAGS). Samples were “spiked” with a Tl standard (^203^Tl-^205^Tl isotopes) to correct for mass-dependent isotopic fractionation. Blank of Pb of the procedure was 0.14 ppb. The entire procedure was monitored using standards SRM-981, and multiple analyses (n = 6) of SRM-981 yielded ^206^Pb/^204^Pb of 16.9390 (σ = 0.0010), ^207^Pb/^204^Pb of 15.4912 (σ = 0.0012) and ^208^Pb/^204^Pb of 36.7148 (σ = 0.0022). Analyses on standard BCR-2 (reference values of 18.750, 15.615 and 38.699 for ^206^Pb/^204^Pb, ^207^Pb/^204^Pb and ^208^Pb/^204^Pb, respectively) resulted in a ^206^Pb/^204^Pb ratio of 18.7573 ± 11 (2σ, *n* = 3), a ^207^Pb/^204^Pb ratio of 15.6246 ± 16 (2σ, *n* = 3) and a ^208^Pb/^204^Pb ratio of 38.7132 ± 12 (2σ, *n* = 3). The BHVO-2 was measured as an external standard (reference values of 18.625, 15.524 and 38.245 for ^206^Pb/^204^Pb, ^207^Pb/^204^Pb and ^208^Pb/^204^Pb, respectively) and yielded a ^206^Pb/^204^Pb ratio of 18.6233, a ^207^Pb/^204^Pb ratio of 15.5261 and a ^208^Pb/^204^Pb ratio of 38.2654.

Hf isotopic data were obtained using a Neptune plus (Thermo Finnigan) multi-collector inductively coupled plasma mass spectrometer (MC-ICP-MS) at Nanjing University. 100 mg powders were leached for 12 h in warm 2.5 N HCl, and dissolved in 15 ml Teflon beakers in an HF-HClO_4_ acid mixture at 120 °C for more than 5 days. After evaporation to dryness, all samples were dried at 200 °C in order to break CaF bonds. Finally, the samples were dissolved in 3 N HCl. Hafnium was separated from the rock matrix by ion exchange procedures using Eichrom® Ln-Spec resin. The detailed analytical procedure for the Hf isotopic measurement can be seen elsewhere^40^. Hf isotopic ratios were normalized to ^179^Hf/^177^Hf = 0.7325. The results were then normalized to a ^176^Hf/^177^Hf value of 0.282160 using the daily average of the JMC 475 Hf standard. The JMC 475 Hf standard analyzed over the period of the analyses gave an average value of ^176^Hf/^177^Hf = 0.282157 ± 0.000005. In addition, international standards BHVO-2 and BCR-2 were also tested in this method. Measured values for BHVO-2 and BCR-2 were 0.283082 ± 0.000004 and 0.282857 ± 0.000006, respectively (Reference values are 0.283101 ± 0.000026 and 0.282867 ± 0.000018, respectively^41^).

### Re-Os isotope analyses

Re-Os isotope analyses were performed in the National Research Center for Geoanalysis, Chinese Academy of Geological Sciences (NRCG-CAGS). Detailed procedures of chemical separation for Re-Os can be referred to Du *et al.*^42^ and Li *et al.*^43^,^44^. Samples were loaded in a Carius tube through a thin neck funnel. The mixed ^190^Os and ^185^Re spike solutions and 3 mL HCl and 6 mL HNO_4_ were loaded while the bottom of the tube was frozen at −50 °C to −80 °C in an ethanol-liquid nitrogen slush with the top sealed by an oxygen-propane torch. The tube was then heated for 24 h at 230 °C. The bottom part of tube was kept frozen during the cooling. The Os was separated through distillation from carius tube for 50 min and was trapped in 5 mL 1:1 HBr. Micro distillation was used for N-TIMS (Triton) determination of Os isotope ratio. The residual Re-bearing solution was saved in a 150 mL Teflon beaker for Re separation.

The residual Re-bearing solution was heated to near-dryness. Then 10 mL of 50% NaOH were added to the residue followed by Re extraction with 10 mL of acetone in a 120 mL Teflon funnel. The acetone phase was transferred to a 100 mL beaker that contains 1 mL water. It was evaporated to dry and picked up in 2% HNO_3_ that was used for the N-TIMS determination of Re isotope ratio. The purified Os and Re were loaded to Pt filaments respectively, and analyzed via negative ion thermal ionization mass spectrometry (NTIMS) using a second electron multiplier (SEM) in peak-hopping mode for Os and by static Faraday collectors for Re. The Re and Os isotope ratios were corrected for mass fractionation using ^185^Re/^187^Re = 0.59738, and ^192^Os/^188^Os = 3.08271. The concentrations of Re and Os were determined by isotopic dilution method, while Os was calculated based on the concentration and natural abundance of ^188^Os in Os. The total procedural blanks of this study are approximately 0.27 ± 0.01 ρg for Os, 0.93 ± 0.04 ρg for Re, and ^187^Os/^188^Os of the blank is 0.205 ± 0.054. The standard material, GBW04477 (JCBY, a net-textured sulfide ore from Jinchuan Cu-Ni sulfide deposit, China), was used to monitor the accuracy of the method. The Re and Os contents of the JCBY determined are 38.28 ± 0.11 ppb and 16.12 ± 0.05 ppb, respectively, which are within uncertainty of the reference values (Re = 38.61 ± 0.54 ppb, Os = 16.23 ± 0.17 ppb).

## Additional Information

**How to cite this article**: Zhang, G.-L. and Li, C. Interactions of the Greater Ontong Java mantle plume component with the Osbourn Trough. *Sci. Rep.* **6**, 37561; doi: 10.1038/srep37561 (2016).

**Publisher’s note:** Springer Nature remains neutral with regard to jurisdictional claims in published maps and institutional affiliations.

## Supplementary Material

Supplementary Information

Supplementary Table
