# Supplementary material for: Interactions of the Greater Ontong Java mantle plume component with the Osbourn Trough
Source: Sci Rep. 2016 Nov 21;6:37561. doi: 10.1038/srep37561 (PMC5116616; doi:10.1038/srep37561)
Supplement: Supplementary Information [file srep37561-s1.pdf]

Interactions of the Greater Ontong Java mantle plume component with the Osbourn Trough

Guo-Liang Zhang[1,2*], Chao Li[3†]

[1] Key Laboratory of Marine Geology and Environment, Institute of Oceanology, Chinese Academy of Sciences, Qingdao 266071, China

[2] Laboratory for Marine Geology, Qingdao National Laboratory for Marine Science and Technology, Qingdao, 266061, China

[3] Institute of Geology Chinese Academy of Geological Sciences, Beijing 100037, China

[*] Corresponding author. E-mail: zhangguoliang@qdio.ac.cn

[†] Corresponding author. E-mail: re-os@163.com.

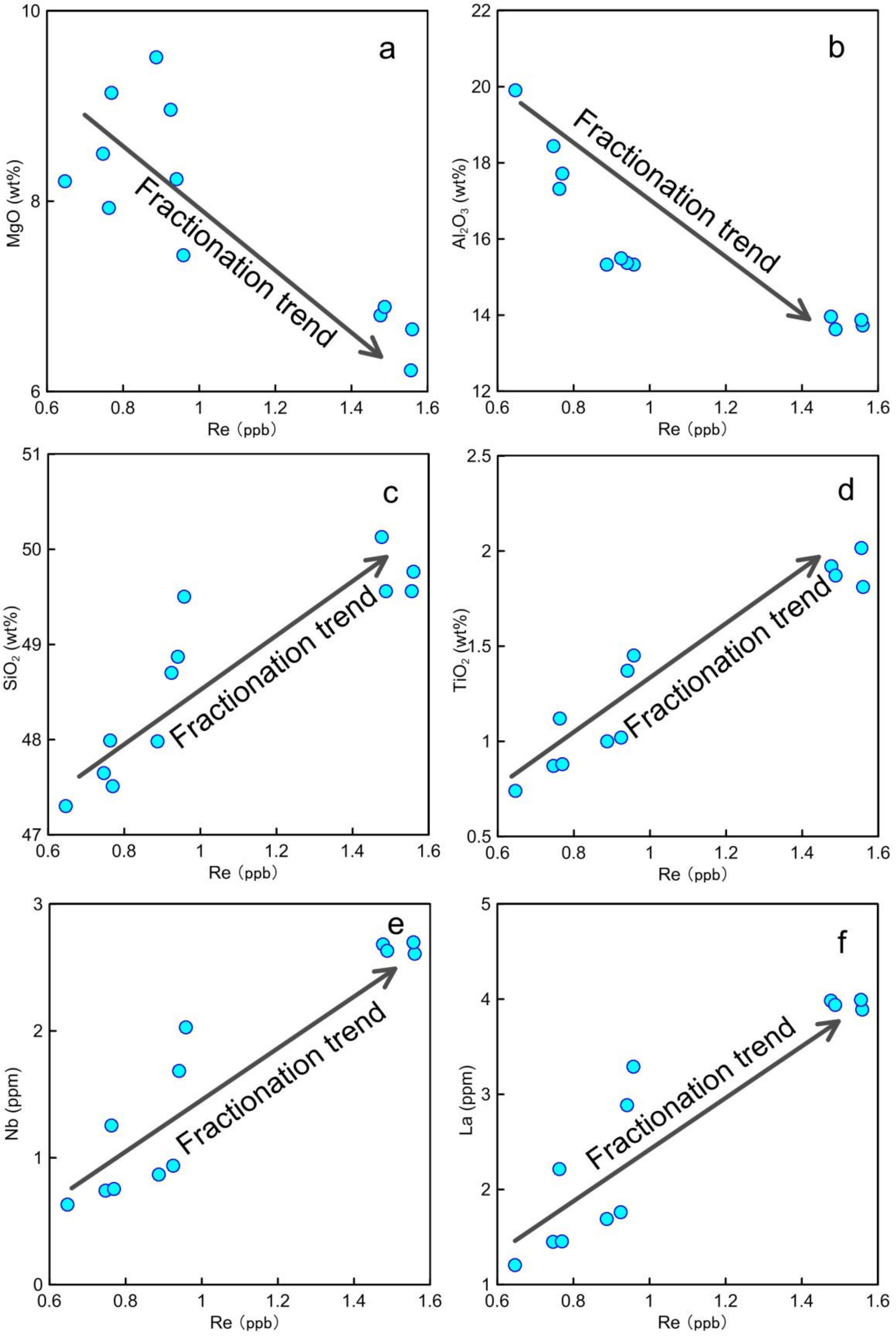

Figure S1 Plots showing relationships of Re with (a) MgO, (b) Al_2O_3, (c) SiO_2, (d) TiO_2, (e) Nb and (f) La.

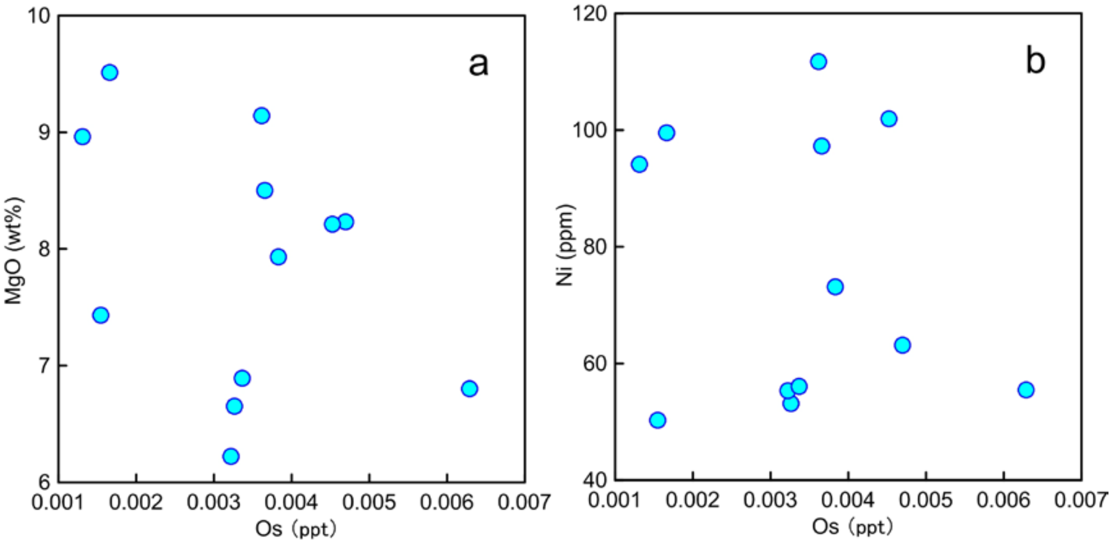

Figure S2. Plots showing relationships of Os vs. (a) MgO and (b) Ni.
